# Exploring patient safety culture and opportunities for improvement: a mixed-methods study in a Dutch paediatric intensive care unit

**DOI:** 10.1136/bmjoq-2025-003571

**Published:** 2025-10-31

**Authors:** Kajal U D Autar, Ada van den Bos-Boon, Gwen G M van Heesch, Monique van Dijk, Marten J Poley

**Affiliations:** 1 Department of Neonatal and Pediatric Intensive Care, Erasmus MC Sophia, Rotterdam, The Netherlands; 2 Erasmus School of Health Policy & Management, Erasmus University Rotterdam, Rotterdam, The Netherlands; 3 Department of Pediatric Surgery, Erasmus MC Sophia, Rotterdam, The Netherlands

**Keywords:** Patient safety, Safety culture, Quality improvement, Paediatrics

## Abstract

**Background:**

Hospitals often face complex and life-threatening situations that heighten the risk of medical errors. Improving patient safety culture is important to reduce these errors. This study aims to identify trends in patient safety culture within a paediatric intensive care unit (PICU) and to explore strategies for improvement.

**Methods:**

The study had a mixed-methods design, combining quantitative and qualitative methods, and was done at the PICU of Sophia Children’s Hospital (Rotterdam, The Netherlands). The Safety Attitudes Questionnaire (SAQ) was used to measure patient safety culture, with surveys administered in 2009, 2012, 2014, 2017, 2019 and 2023. Trends in patient safety culture over time were analysed. Additionally, staff members provided recommendations to improve patient safety, which were subsequently categorised into overarching themes. An expert panel was convened and interviews with staff members were conducted to further evaluate the most frequently mentioned recommendations and assess their relevance and feasibility for implementation.

**Results:**

From 2009 to 2023, patient safety culture demonstrated overall improvement. However, specific domains, including stress recognition, perceptions of management and working conditions, still show room for further improvement. Most recommendations identified through the SAQ fell within the themes of interprofessional communication, medical equipment and hospital working environment, and staffing. Concrete suggestions included appointing a dedicated contact person to improve communication with parents and establishing clear agreements to strengthen communication and teamwork within the PICU.

**Conclusions:**

The patient safety culture at the PICU of Sophia Children’s Hospital improved over the years, although areas for improvement remain. Sustained improvements in patient safety culture require continuous investment in interprofessional communication, workplace conditions and staffing. This study not only highlights long-term trends but also presents actionable strategies proposed by staff to address persistent challenges. Effective implementation and ongoing evaluation of these interventions are essential to strengthen safety culture, enhance staff well-being and ultimately improve patient outcomes.

**WHAT IS ALREADY KNOWN ON THIS TOPIC**

- Patient safety culture plays a vital role in reducing medical errors and in improving the overall quality of healthcare. Previous studies showed that the safety culture in paediatric intensive care units (PICUs) is often suboptimal, leaving room for improvement. Measuring patient safety culture is a critical first step in addressing this issue, and validated tools such as the Safety Attitudes Questionnaire (SAQ) are available for this purpose.

**WHAT THIS STUDY ADDS**

- Using a combination of methods, including the SAQ, this study provided a 14-year longitudinal analysis of patient safety culture trends in a PICU, and analysed numerous staff recommendations for improving patient safety. Overall, patient safety culture in the PICU gradually improved over the years, but the study also highlighted specific domains that require further attention. The findings included practical and feasible suggestions for improving patient safety culture.

**HOW THIS STUDY MIGHT AFFECT RESEARCH, PRACTICE OR POLICY**

- This study can serve as a foundation for future research on (gaps in) patient safety culture in PICUs and on how staff recommendations can be effectively implemented to achieve optimal results. The findings may influence the development of patient safety protocols in PICU settings, which in the end will contribute to a stronger overall safety culture and improved patient outcomes.

## Introduction

 In hospitals, complex and life-threatening situations frequently arise. An estimated 98 000 people die each year due to medical errors, such as surgical injuries or mistaken identity.^1^ A more recent estimate suggests that approximately 200 000 preventable hospital-related deaths occur annually in the USA.^2^ Many of these errors, often caused by communication breakdowns or deficits in clinical performance, are preventable. Addressing this issue requires improving the patient safety culture, which is about the values, norms and behavioural characteristics of staff members in relation to their organisation’s patient safety performance.^3^

Monitoring patient safety culture over time can help identify areas for improvement. Several tools have been developed to measure patient safety culture from the perspective of frontline workers, including the Safety Attitudes Questionnaire (SAQ)^4^ and the Hospital Survey on Patient Safety Culture.^5^ Despite the growing use of these instruments, evidence on the patient safety culture in paediatric healthcare settings remains limited. Studies suggest that the safety culture in these settings is often suboptimal, leaving room for improvement.^6 7^

Moreover, there is little information on practical approaches to improving patient safety culture. A few studies, using the SAQ, analysed staff recommendations for improvement. For example, recommendations in the study by Gleeson *et al* included ‘staffing issues’, ‘patient-focused care’, ‘hospital environment’, ‘safe reporting culture’ and ‘training and education’.^8^ An example of a recommendation from the study by Campione and Famolaro was ‘action planning where results are analysed and shared with staff’.^9^ These studies suggest various strategies to enhance patient safety culture, including appointing a case manager to improve communication between medical staff and patients and promote unity among staff members^10^ and implementing feedback interventions to enhance communication and interdependence among staff.^11^

A previous SAQ-based study done in our paediatric intensive care unit (PICU) at Sophia Children’s Hospital found that, while patient safety culture was strong compared with benchmark data, there was room for improvement.^7^ Staff recommended actions such as ‘improve nurses-physician communication’ and ‘report and take action on incidents, complications and deaths’. Since then, several initiatives have been introduced in our PICU, including structured debriefings and enhanced incident reporting mechanisms. However, challenges in communication and incident follow-up remain. This suggests that, despite the progress that has been made, further efforts are needed to ensure sustained improvements in patient safety culture.

Therefore, this study aims to (1) identify trends in patient safety culture over time within our PICU, as measured by the SAQ and (2) identify areas where patient safety could be improved, based on staff recommendations collected through the SAQ and subsequently assessed for relevance and feasibility by an expert panel and discussed in staff interviews.

## Methods

### Study design

The study design was a combination of a retrospective and a prospective study, using both quantitative and qualitative methods. The quantitative component was retrospective, using SAQ data collected since 2009. The qualitative and prospective components of the study consisted of an expert panel and staff interviews. Patients or the public were not involved in the design, conduct, reporting or dissemination plans of our research.

### Study setting

The setting of this study was the PICU at a tertiary university children’s hospital in an urban environment, serving a referral area of approximately 5 million people (constituting >25% of the Dutch population) with 44 000 births each year. The PICU has a capacity of 28 beds, distributed across four units: two intensive care units and two high care units. It admitted 1383 patients in 2023, ranging in age from newborn to 22 years (56% below the age of 3 years). The PICU offers comprehensive care across all paediatric and surgical subspecialties.

### Data sources, data collection and data analysis

#### Safety Attitudes Questionnaire

The study used the SAQ to collect data on patient safety culture.^4^ The SAQ is a widely used tool, which consists of 36 items that evaluate six dimensions of patient safety culture: teamwork climate, job satisfaction, perceptions of management, safety climate, working conditions and stress management. All items are answered using a 5-point Likert scale, ranging from disagree strongly (score of 0), disagree slightly (25), neutral (50), agree slightly (75), to agree strongly (100). Each scale score was calculated as the mean of its component items and thus was calibrated from 0 to 100. No scale score was calculated if any of the component items were missing. Negatively worded items were reverse scored, so, for all scales, higher scores indicated a better patient safety culture. The final section of the SAQ asks respondents to give their top three recommendations to improve patient safety in their department. Several studies showed that the SAQ is valid and reliable.^412^,^14^ The SAQ used in this study was translated into Dutch using a standardised forward-backward procedure.^7^

The SAQ was administered in 2009, 2012, 2014, 2017, 2019 and 2023. All staff members, both full-time and part-time, with a significant work attendance in the PICU, were eligible to participate. This included physicians, nurses, nurse practitioners, pharmacists, technicians, ward clerks and managers. The questionnaires were handed out during crew resource management (CRM) training and completed anonymously.

The data analysis began by examining the trend in patient safety culture over time. Descriptive statistics were used to show how the six subscale scores of the SAQ varied across survey years. Second, all provided recommendations were categorised into the following twelve themes, which are described in table 1:

- Staffing.
- Medical equipment and hospital working environment.
- Training (and education).
- Incident reporting.
- Action planning.
- Communication between professions.
- Feedback.
- Teamwork.
- Communication with parents.
- Protocols.
- Working conditions.
- Continuity of care.

**Table 1:** Descriptions of themes

Theme	Description
Staffing	Staff shortages, qualified staff and occupation according to care level.
Medical equipment and hospital working environment	All equipment and their usage that are important for the care processes.
Training (and education)	All training and forms of education, including scenario training, clinical lessons about illnesses, education about the use of medical equipment, CRM training and the training and supervision of interns.
Incident reporting	Everything that is related to reporting of (near) incidents or quality issues and to the provision of feedback following reporting of incidents.
Action planning	All steps guiding the organisation to focus on patient safety.
Communication between professions	All sorts of communication between professions, such as physical communication, documentation of patient’s data and meetings, including communication tools such as (de)briefings, huddles and evaluations.
Feedback	All aspects of feedback giving and receiving.
Teamwork	Collaborations within and between professions and work culture.
Communication with parents	All sorts of communication with parents.
Protocols	Stimulating evidence-based ways of working, among others by the use of protocols and keeping protocols up to date.
Working conditions	Different aspects of the job, such as way of working and work hours.
Continuity of care	Continuity in medical policy and staff, including devoting sufficient attention and time to the patient.

CRM, crew resource management.

The first six of these themes were taken from previous studies that analysed recommendations from the SAQ.^7^,^9^ Six additional themes emerged from frequently mentioned recommendations in this study. While some themes are related (eg, communication between professions, feedback and teamwork), they were categorised separately, as each was identified as a distinct and important issue by the respondents. A second reviewer independently assessed cases where there was uncertainty about the categorisation of a particular recommendation. Any disagreements between the two reviewers were resolved through consensus, with input from a third reviewer when necessary.

Once each recommendation was assigned to a theme, it was counted how many times a theme was mentioned for each survey year and for the full sample. Then, it was analysed which specific recommendations were most frequently mentioned under those themes each year. The analysis resulted in a final list of themes and recommendations, which was used for both the expert panel and the interviews.

#### Expert panel

An expert panel was held to evaluate the most mentioned recommendations. Practically, this implied that the recommendations that were considered irrelevant (or no longer relevant) were excluded. The expert panel also discussed whether and how the recommendations could be implemented to see if implementation was feasible. The expert panel consisted of a manager, a senior nurse and a physician, all actively involved as leaders in patient safety tasks and therefore recognised as patient safety experts. They were selected from our PICU staff based on their extensive work experience within our unit and their acknowledged expertise in patient safety in paediatric intensive care.

#### Interviews

In the interviews, staff members were asked for ideas on how to implement the recommendations for improving patient safety. A total of 10 interviews were done, among 2 managers, 3 physicians and 5 nurses working at the PICU. A manager at the PICU suggested respondents, who were then randomly selected from different units to ensure a diverse representation of perspectives, minimising the risk of selection bias. The interviews were semistructured and guided by a topic list. They consisted of questions such as ‘What is the situation like now (for a certain theme)?’ and ‘How would you implement the recommendation?’.

Results from the expert panel and the interviews were combined in the analysis due to overlapping responses, allowing for a more efficient presentation of the findings.

For the data analysis, all interviews were recorded and transcribed. The transcriptions were then coded in three stages.^15^ First, during open encoding, codes were connected to the text. Second, during axial encoding, the coded pieces were put below the corresponding themes. Third, during selective encoding, the coded pieces were connected to each other. The themes of the recommendations were used as the codes for the analysis.

## Results

### Patient safety culture in the Sophia Children’s Hospital

#### Descriptive statistics

The number of respondents who completed the SAQ ranged from 153 to 191 per survey year, with a median of 175 and a total of 1029 (table 2).

**Table 2:** Background characteristics of respondents across the years

		2009 N=153	2012 N=180	2014 N=178	2017 N=172	2019 N=155	2023 N=191
Gender, n (%)	Male	17 (11.1)	13 (7.2)	14 (7.9)	11 (6.4)	13 (8.4)	16 (8.4)
	Female	136 (88.9)	161 (89.4)	164 (92.1)	159 (92.4)	122 (78.7)	175 (91.6)
	Missing	0 (0.0)	6 (3.3)	0 (0.0)	2 (1.2)	20 (12.9)	0 (0.0)
Age in years	Mean (SD)	40.9 (8.9)	42.1 (9.0)	43.6 (9.4)	45.0 (9.8)	42.5 (10.9)	42.8 (12.1)
Profession, n (%)	Nurse	104 (68.0)	129 (71.7)	123 (69.1)	112 (65.1)	83 (53.5)	121 (63.4)
	Physician	15 (9.8)	15 (8.3)	18 (10.10)	22 (12.8)	14 (9.0)	21 (11.0)
	Other	28 (18.3)	30 (16.7)	31 (17.4)	33 (19.2)	34 (21.9)	49 (25.6)
	Missing	6 (3.9)	6 (3.3)	6 (3.4)	5 (2.9)	24 (15.5)	0 (0.0)
Followed a CRM- training course on this PICU, n (%)	Yes	137 (89.5)	152 (84.4)	143 (80.3)	162 (94.2)	145 (93.5)	132 (69.1)
	No	16 (10.5)	26 (14.4)	15 (8.4)	10 (5.8)	10 (6.5)	59 (30.9)
	Missing	0 (0.0)	2 (1.1)	20 (11.2)	0 (0.0)	0 (0.0)	0 (0.0)
Employment, n (%)	Full-time	60 (39.2)	55 (30.6)	52 (29.2)	56 (32.6)	55 (35.5)	70 (36.6)
	Part-time	92 (60.1)	112 (62.2)	121 (68.0)	110 (64.0)	78 (50.3)	120 (62.8)
	Flex work	1 (0.7)	9 (5.0)	4 (2.2)	2 (1.2)	1 (0.6)	1 (0.5)
	Missing	0 (0.0)	4 (2.2)	1 (0.6)	4 (2.3)	21 (13.5)	0 (0.0)

CRM, crew resource management; PICU, paediatric intensive care unit.

Most of the respondents were females, with an average age in their early 40s, and most were employed part-time. Furthermore, most respondents had followed a CRM training. Nurses were clearly overrepresented. Generally, the distribution of these characteristics was quite stable throughout the study years.

#### Trends over time

**Figure 1:**
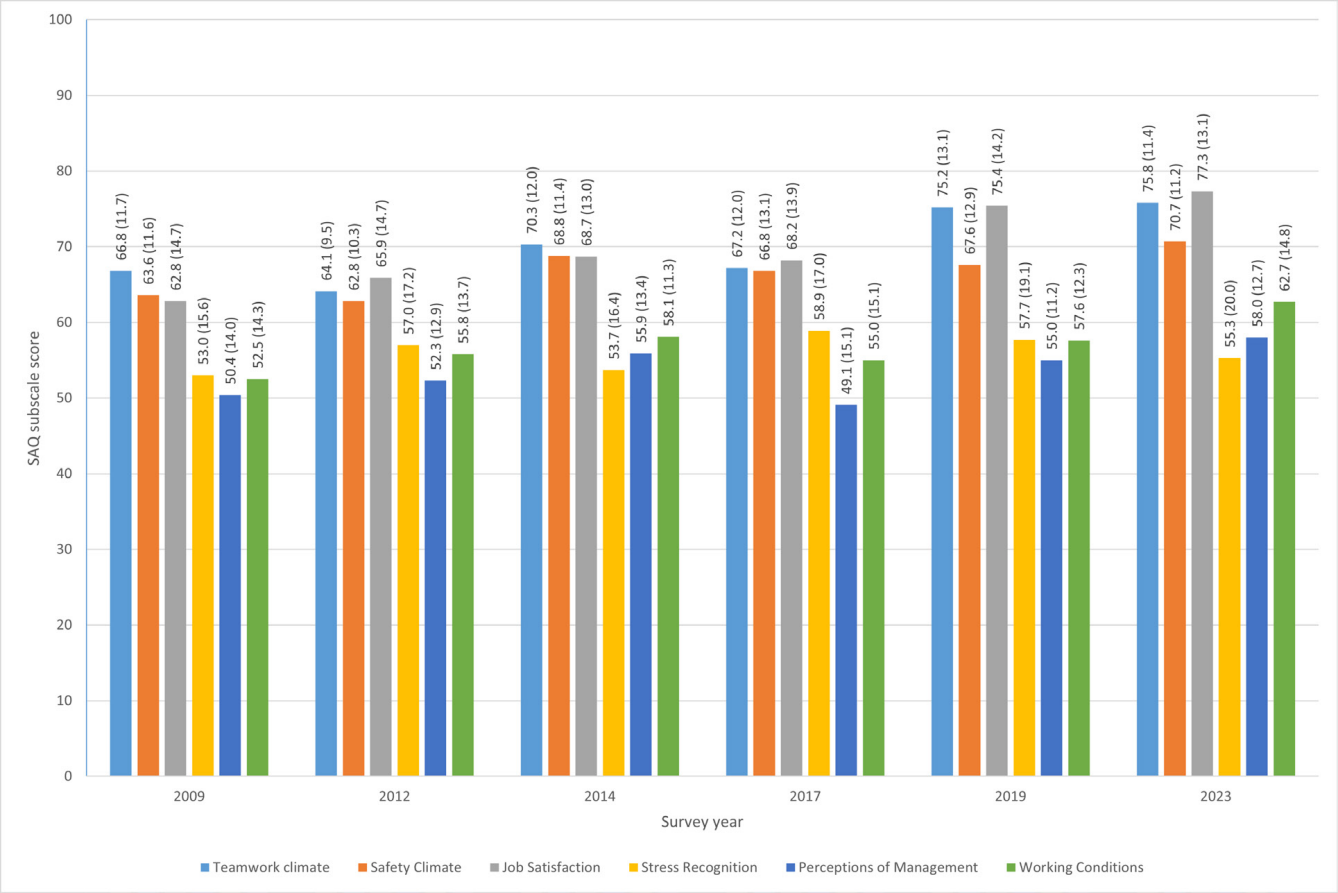
Mean SAQ subscale scores (with SD) per survey year. SAQ, Safety Attitudes Questionnaire.

Figure 1 presents the mean SAQ subscale scores (with SD) throughout the survey years. Teamwork climate, job satisfaction and safety climate consistently had the highest scores. Generally, the subscale scores appeared to increase over time, although some years, particularly 2017, showed interruptions in this positive trend. When comparing 2023 to 2009, most scale scores increased, with medium effect sizes (Cohen’s d ranging from 0.57 to 0.77). Job satisfaction was an exception, showing a relatively large increase (77.3 vs 62.8; Cohen’s d=1.05). For stress recognition, the score in 2023 was only 2.3 points higher than in 2009, indicating a small effect size (Cohen’s d=0.13). Furthermore, the subscale scores of 2023 show that there is still room for improvement, especially in stress recognition, perceptions of management and working conditions, where the scores are below 70. The recommendations from the SAQ are analysed in the next section to explore options for improving the patient safety culture in our PICU.

### Recommendations to improve patient safety culture

**Figure 2:**
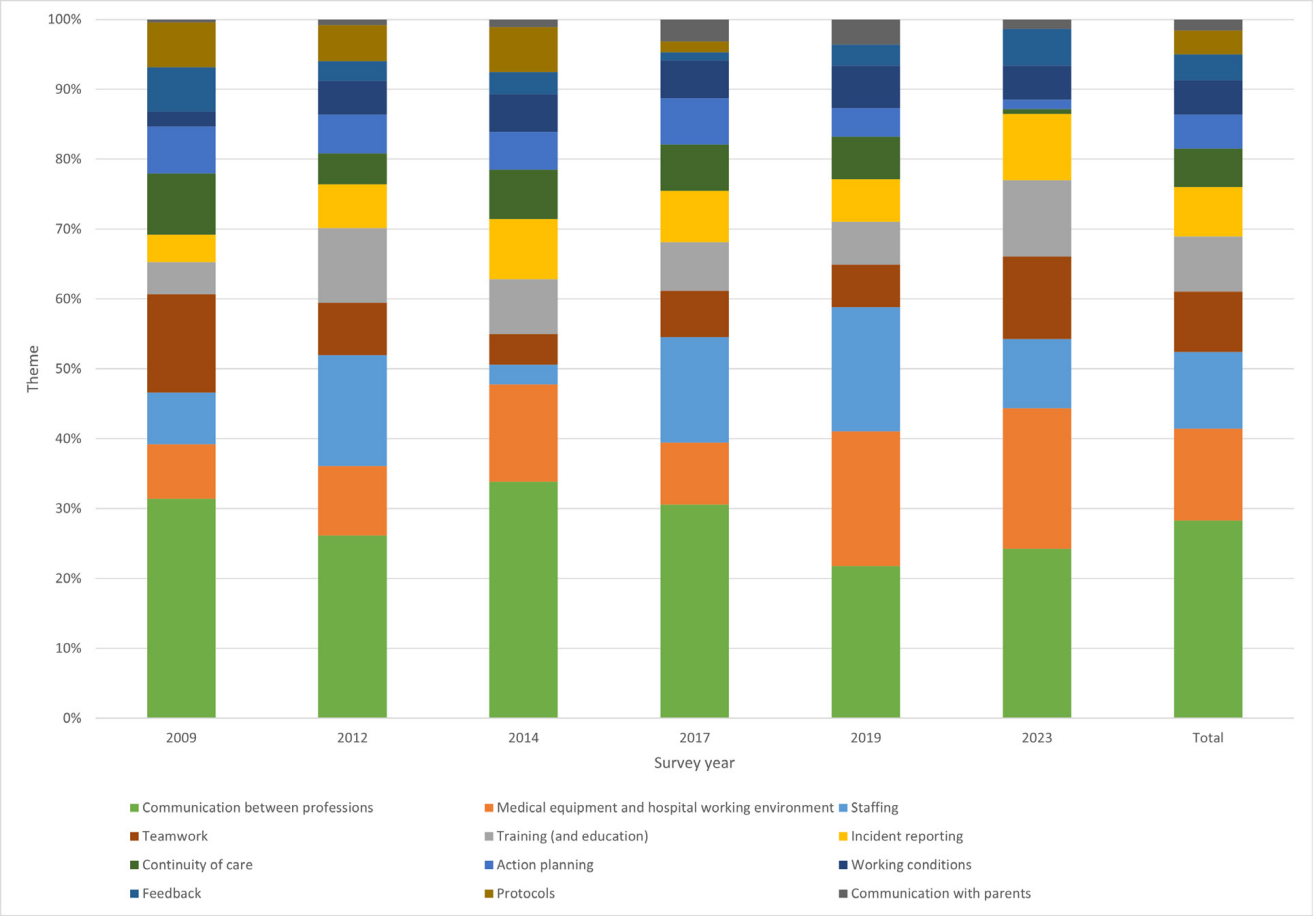
Recommendations per theme (per survey year and full sample).

Out of the 1029 respondents, 705 provided a total of 1574 recommendations. Figure 2 shows that most of these recommendations fell within the themes of communication between professions (28% of all recommendations), medical equipment and hospital working environment (13%) and staffing (11%). Throughout the years, these frequently mentioned themes were rather stable, showing their ongoing relevance. However, the theme of ‘medical equipment and hospital working environment’ seemed to increase in recent years, while ‘staffing’ showed quite some variation over time. Table 3 shows which specific recommendations were most mentioned under those themes.

**Table 3:** Most mentioned recommendations

Theme	Most frequently mentioned recommendations
Staffing	‘More staff’, ‘better occupation according to the care level’, ‘more staff next to the bed’ and ‘more qualified nurses’.
Medical equipment and hospital working environment	‘More workspace’, ‘better availability of materials’, ‘better implementation of medical equipment’, ‘better equipment for medication monitoring’, ‘double checking of medications’ and ‘medication administration checks’.
Training (and education)	‘More CRM training’, ‘more clinical lessons about illnesses’ and ‘more scenario training’.
Incident reporting	‘Easier way to report incidents’, ‘more incident reporting’, ‘more feedback/discussion/evaluation of incident reports’.
Action planning	‘Transparency and sharing results and improvements of the PICU with staff members’.
Communication between professions	‘Better communication between disciplines and other departments’, ‘better documentation in HiX (patient documentation system)’, ‘better medical information transfers’, ‘more use of briefings’, ‘more use of huddles’, ‘more debriefings during and after complicated cases’ and ‘more use of debriefings after acute situations’.
Feedback	‘More giving and receiving of feedback between staff members on the work floor’.
Teamwork	‘Better teamwork between disciplines’, ‘more respect for each other’, ‘more team spirit’ and ‘open and safe team culture’.
Communication with parents	‘Better communication with parents’.
Protocols	‘Work according to protocol’, ‘evidence-based working’, ‘more accessible protocols’ and ‘up-to-date protocols’.
Working conditions	‘Better work hours’, ‘work pressure’ and ‘rules regarding absence of staff members on the PICU’.
Continuity of care	‘Having the same physician/nurses for a patient’, ‘more similar medical policy by different physicians’, ‘keeping the patient on the same unit’, ‘better after care’ and ‘more time for the patient’.

CRM, crew resource management; PICU, paediatric intensive care unit.

### Relevance, feasibility and implementability of the recommendations to improve patient safety culture

The relevance and feasibility of the recommendations were assessed by the expert panel. For each theme deemed relevant and feasible, explanations and potential solutions for implementing the recommendations are given. The findings of the expert panel and those of the interviews with staff members often overlapped, which is why they are discussed together in this section. In the presentation of these results, some related themes will be combined: staffing and working conditions, incident reporting and action planning, and communication between professions, feedback and teamwork.

#### Staffing and working conditions

Increasing staffing levels is relevant, but implementation is complex due to financial constraints. However, optimising staff allocation based on care intensity is already a priority. The management is already in conversation with staff members on how to reduce work pressure. Minor adjustments can be made to further address this issue. For example, introducing clearer rules regarding staff absences from the PICU due to other tasks could help reduce work pressure.

#### Medical equipment and hospital working environment

The experts mentioned that availability of materials is currently an issue, because of shortages of original materials. Therefore, substitute materials are being used now. Issues concerning workspace and materials are already recognised by the management. Solutions will be included in the planned renovation of the hospital.

#### Training (and education)

The experts emphasised that more priority should be given to all sorts of training, considering it a management decision. “Staff members should also initiate training themselves” (Expert 1). However, not all staff members are doing this. Managers have implemented a new training plan that includes a points system where staff members must accumulate a certain number of points by the end of the year. Points can be earned through activities such as clinical lessons and theme evenings. This point system encourages continuous professional development and helps identify areas where additional training may be needed.

#### Incident reporting and action planning

Incident reports are analysed monthly by a team that focuses on quality. Feedback from these analyses is shared with staff through a monthly newsletter. However, according to the expert panel, there is a desire for greater insight and feedback regarding the outcomes of incident reporting. This sentiment was shared by the interviewed nurses, one of whom stated: “Rather than only sharing the big things, it is also important to share the little things” (Nurse 4). This approach could foster a more engaged and informed culture, as staff would better understand how their reports are being used.

Current initiatives, such as newsletters with concise summaries and screensavers, are valuable. Additionally, Physician 1 suggests: “One or two education days on incident reporting might be useful. Visualize what was reported in the last year, what has improved. And make visible who is part of the quality team, how the system works, and be open to ideas from the staff”. This could enhance satisfaction with the incident reporting process.

#### Communication between professions, feedback and teamwork

The expert panel identified variability in communication between professions. Expert 2 mentions: “As a nurse, you don’t know that the physician came in because they didn’t inform us. What can be improved is the live communication”. Expert 3 adds: “Parents also mention that they hear something from one physician, but the other doesn’t know anything about that. This can be frustrating for the parents if they have to repeat things”. The expert panel believed that the implementation of recommendations regarding communication between professions could be further developed. Interviewees noted that consultant physicians, who are not always present on the PICU, sometimes visit the unit to speak with parents without informing staff. As a result, the responsible nurse may be unaware of what was discussed. Additionally, as one physician mentioned: “This miscommunication leads to confusion and disappointment if things turn out differently when for example other decisions are made” (Physician 2).

A solution could be to establish clear agreements about how consultant physicians should communicate when entering the unit. All respondents agreed that the consultant physician should notify the nurse, senior nurse or physician before or after a visit. Nurse 1 mentions that consultant physicians could say the following to a staff member if the responsible nurse is not available: “Could you say that I was here and discussed this…”. To ensure these agreements are effectively implemented, all staff members have to be aware of them, committed to them and continually communicate them with consultant physicians.

Staff members provided the recommendation to increase the exchange of feedback between staff members on the work floor. The expert panel, however, acknowledged that feedback is challenging, because it is ingrained in the culture, which takes time to evolve and involves many components. It encompasses example behaviour, psychological safety and how feedback is handled (Expert 3). Expert 1 notes: “Staff members don’t find it difficult to address things, but the way they address things can be improved. Sometimes there is too much emotion or responses are too blunt. Feedback has to do with the tone, the way things are said”. Providing feedback is a crucial element of a patient safety culture, yet it is not well-defined how strategies for improving the feedback process can be implemented.

Manager 1 mentions: “In general, there is psychological safety on the unit to give feedback”. However, Nurse 2 remarks: “Psychological safety could be better”. The interviewees suggest that increasing feedback and psychological safety can be achieved by becoming accustomed to giving and receiving feedback, even on minor issues. It is essential to address matters in an approachable way and make the other person aware of how their actions made you feel.

#### Communication with parents

A frequently mentioned recommendation was to increase the involvement of patients’ parents. Expert 3 remarked: “More involvement of parents will lead to greater satisfaction of both parents and patients. It will also improve the current way of working and teamwork. Some staff members believe that involving parents is difficult due to the complexity of the PICU. However, this perception is unfounded. There are various levels and ways to involve parents”. The expert panel felt that it is not yet clear how to implement the recommendations regarding communication with parents. One issue identified is the variability among physicians. “If parents talk with different physicians, they hear different stories because everyone expresses themselves differently. This can be frustrating if you are already in a stressful situation” (Physician 3).

The interviewees suggested assigning a key role to the nurse practitioner to improve communication between physicians and parents. “The nurse practitioner has more regular shifts and serves as the dedicated contact person for parents” (Physician 3). Nurse 2 adds: “It would be nice if there would be a case manager for the parents, where they can ask different types of questions. This person knows the family better and can be a frequent contact person”.

#### Protocols

The recommendations highlight the importance of clear and up-to-date protocols to ensure consistency and quality of care. Although the experts acknowledged challenges in maintaining protocols—often due to a lack of dedicated responsibility—they emphasised that protocols remain essential for promoting evidence-based practices. Ongoing efforts focus on improving the development and regular updating of protocols to better support staff in delivering safe and standardised care.

#### Continuity of care

The recommendations highlight inconsistencies in medical policies among different physicians as a potential obstacle to patient safety. Expert 1 notes that physicians are already working to address this issue. Physician 2 mentions that whenever possible, the same physicians and nurses are assigned to long-stay PICU patients. Expert 3 mentions that this approach provides greater continuity of care.

## Discussion

This study assessed patient safety culture in the PICU at Sophia Children’s Hospital by identifying key areas for improvement based on staff feedback. Although patient safety culture has improved over the years, further progress can be achieved by focusing on the recommendations highlighted in the SAQ.

Staff in this study provided numerous recommendations through the SAQ. The themes emerging from these recommendations aligned closely with those found in earlier studies.^7 8^ For example, in the study of Gleeson *et al*, two major themes were ‘staffing issues’ and ‘hospital environment’,^8^ which is consistent with our findings, where ‘staffing’ and ‘medical equipment and hospital working environment’ were the most frequently mentioned themes. This suggests that common patient safety issues occur across clinical settings. However, ‘teamwork’, a frequently mentioned theme in our study, was less prominent in prior studies, marking a notable difference.

The recommendations from the SAQ offered insights beyond those that can be derived from SAQ scale scores alone. After all, while some recommendations addressed issues reflected in low SAQ scale scores (eg, staffing issues and the SAQ scale ‘working conditions’), others highlighted concerns that were not matched by low SAQ scale scores (eg, communication issues and the SAQ scale ‘teamwork’). Some recommendations were vague—such as ‘better communication’ or ‘better teamwork’—and thus provided few actionable cues for improvement. The expert panel and interviews helped refine these suggestions into concrete actions. These qualitative insights clarified staff needs and identified practical strategies to strengthen patient safety culture.

Solutions from the literature and this study’s interviews to improve patient safety culture showed similarities. First, during the interviews, it was suggested to appoint a nurse practitioner or staff member as a ‘dedicated contact person’ or ‘case manager’ to improve communication with parents. This corresponds with earlier literature that highlighted the benefits of case management in improving communication and increasing satisfaction among patients and their families.^10^

Second, respondents mentioned that consultant physicians sometimes visit the unit to speak with parents without informing staff members. A proposed solution was to establish a protocol requiring staff to be notified before or after the consultant physician meets with parents. This suggestion aligns with Chapman’s recommendation to notify the nurse responsible for the patient on the physician’s arrival on the unit.^16^

Third, staff expressed a desire for greater insights into incident reporting trends, such as through education days. Alternatively, instead of education days, brief discussions during weekly handovers could offer a practical, continuous learning approach, given staff shortages that may hinder attendance at education days. Enhancing awareness of incident reporting trends could boost staff engagement, ultimately improving the reporting system. This aligns with quality monitoring strategies described by Powell *et al*^17^

Fourth, feedback emerged as an important topic in this study. Respondents emphasised the need to improve psychological safety by fostering an environment where concerns are addressed openly and where individuals communicate how they feel. This aligns with the concept of ‘graded assertiveness’, which involves using gentle cues, such as “I am concerned about…” and “It would help me to understand…”, to express concerns.^18^ While previous studies have highlighted the value of socially constructed learning through peer dialogues,^11^ our findings provide additional practical insights by revealing specific barriers and preferences within the PICU context. For example, although written feedback is currently used, staff expressed a strong preference for live, real-time feedback to foster immediate learning and improve team dynamics. This supports Brehaut *et al*’s suggestion for real-time feedback,^11^ but extends it by highlighting its relevance in high-stakes environments like PICUs, where timely communication is crucial.

### Strengths and limitations

A strength of this study was the use of informal member checking during interviews, allowing participants to confirm or correct summarised statements. Reliability was ensured through the maintenance of an audit trail, documenting all steps and decisions throughout the research process. Additionally, data collection followed a structured sequence: SAQ data analysis was conducted first, followed by an expert panel review, and finally, interviews. This ensured that the interviews were informed by both prior data and expert insights. The validity of the study was supported by using the SAQ, a widely used tool with strong psychometric properties. Furthermore, a high response rate was achieved by having staff complete the SAQ immediately before mandatory CRM training sessions. Although exact response rates cannot be reported—because the total number of staff per professional group for each year was unavailable—we are confident that participation represented the large majority of physicians and nurses in each survey year (probably exceeding 80%), making it reasonable to consider the findings representative of the PICU staff perspective.

A potential limitation of the study is the relatively small number of interviews, with ten participants. Given that the number of nurses on the PICU (over 120 in the year 2023) far exceeds the number of nurses interviewed (five), some opinions could have been missed. However, the study included nurses from different units, as well as both senior and junior nurses, which likely helped to capture a broad range of viewpoints. Furthermore, data saturation was reached, as most respondents provided similar statements.

### Future recommendations

Based on the recommendations given through the SAQ, the following actions can be advised. First, communication with parents should be facilitated by assigning a dedicated contact person for each patient. Second, to enhance communication and teamwork between professions, stronger agreements on how to act and communicate within the PICU should be implemented. Given the dynamic nature of PICU teams and ongoing staff turnover, this requires continuous attention, reflection and adaptability. Regular reflection moments and the integration of learning into daily routines can help teams adjust to changes and maintain effective collaboration. Third, for incident reporting, visualisation of reports by education days could be implemented. Fourth, further strategies to enhance feedback are recommended.

While some actions will demand significant effort, time and financial investment, this study also identified low-resource strategies. For example, improving live communication between consultant physicians and staff through clear agreements can be achieved relatively easily, although these agreements will require ongoing reinforcement.

Future studies should aim to provide more insights into how patient safety culture in PICUs (and potentially in other paediatric and adult healthcare settings) can be further improved. Elaborate implementation plans should then be developed, guided by the principles of effective change implementation outlined by Grol *et al.*^19^ Afterwards, it should be evaluated whether patient safety culture has actually improved, through interviews and additional prospective analysis of SAQ results. Furthermore, SAQ results and implemented improvements could be compared across different hospitals to identify general best practices.

### Conclusions

This study highlighted key areas for improvement in the patient safety culture, especially stress recognition, perceptions of management and working conditions. Staff recommendations centred on communication between professions, medical equipment and hospital working environment, and staffing. Proposed strategies to improve the patient safety culture included appointing a dedicated contact person to improve communication with parents and establishing agreements to strengthen interprofessional communication and teamwork. Implementation of these interventions is essential to strengthen the patient safety culture, ultimately leading to better patient outcomes.

## Data Availability

Data are available on reasonable request.
